# Supplementary material for: Pulmonary Manifestations of Plasma Cell Type Idiopathic Multicentric Castleman Disease: A Clinicopathological Study in Comparison with IgG4-Related Disease
Source: J Pers Med. 2020 Dec 10;10(4):269. doi: 10.3390/jpm10040269 (PMC7768369; doi:10.3390/jpm10040269)
Supplement: Supplementary file 1 [file jpm-10-00269-s001.zip › TableS1.docx]

**Supplementary Table S1.** Comparison of computed tomography(CT) findings

|  | PC-iMCD (n=15^†^) | IgG4-RD (n=6^‡^) | *p*-value |
| --- | --- | --- | --- |
|  |  |  |  |
| Location (n,%) |  |  |  |
| All lobes | 14 (93.3) | 6 (100) | 1.000 |
| uppper lobe dominant | 0 | 1 (16.7) | 0.286 |
| lower lobe dominant | 1 (6.7) | 1 (16.7) | 0.500 |
| Localized | 1 (6.7) | 0 | 1.000 |
| CT findings (n,%) |  |  |  |
| round GGO | 12 (80.0) | 4 (66.7) | 0.598 |
| solid nodular | 14 (93.3) | 5 (83.3) | 0.500 |
| Cyst | 4 (26.7) | 1 (16.7) | 1.000 |
| Thickning of BvB | 15 (100) | 5 (83.3) | 0.286 |
| Thickning of Interlobular septa | 15 (100) | 5 (83.3) | 0.286 |
| Pleural effusion | 0 | 0 | NS |
| Differential diagnoses on CT images (n,%) |  |  |  |
| Lymphoproliferative disease | 7 (46.7) | 1 (16.7) | ― |
| Sarcoidosis | 5 (33.3) | 1 (16.7) | ― |
| Interstitial pneumonia | 4 (26.7) | 1 (16.7) | ― |
| Collagen disease | 3 (20.0) | 0 | ― |
| Lymphoma | 3 (20.0) | 0 | ― |
| Bronchial pneumonia | 2 (13.3) | 3 (50.0) | ― |
| Lung cancer | 2 (13.3) | 1 (16.7) | ― |
| Fungal infection | 1 (6.7) | 1 (16.7) | ― |
| Amyloidosis | 1 (6.7) | 0 | ― |
| Pulmonary edema | 0 | 1 (16.7) | ― |
| †, ‡ CT reports were available for 15 PC-iMCD patients and six IgG4-RD patients. Fisher’s exact test was used for the analysis of nominal scales. PC-iMCD, plasma cell-type idiopathic multicentric Castleman disease; IgG4-RD, IgG4-related disease; GGO, ground glass opacity; BvB, bronchovascular bundle; NS, no significant difference. | | | |
