# Supplementary figures and images for: Pulmonary Manifestations of Plasma Cell Type Idiopathic Multicentric Castleman Disease: A Clinicopathological Study in Comparison with IgG4-Related Disease
Source: J Pers Med. 2020 Dec 10;10(4):269. doi: 10.3390/jpm10040269 (PMC7768369; doi:10.3390/jpm10040269)

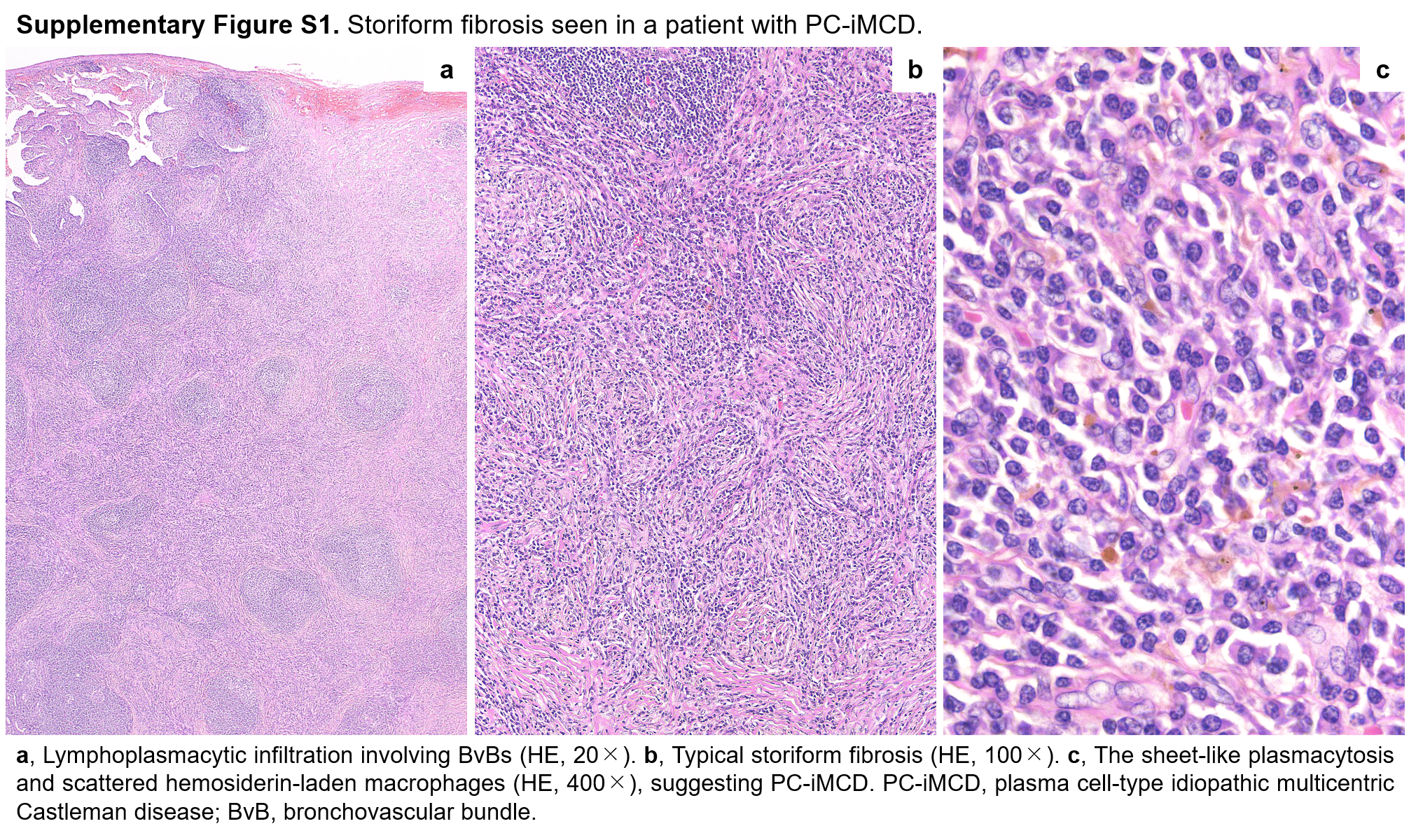

Supplement: Supplementary file 1 [file jpm-10-00269-s001.zip › FigureS1.tif]

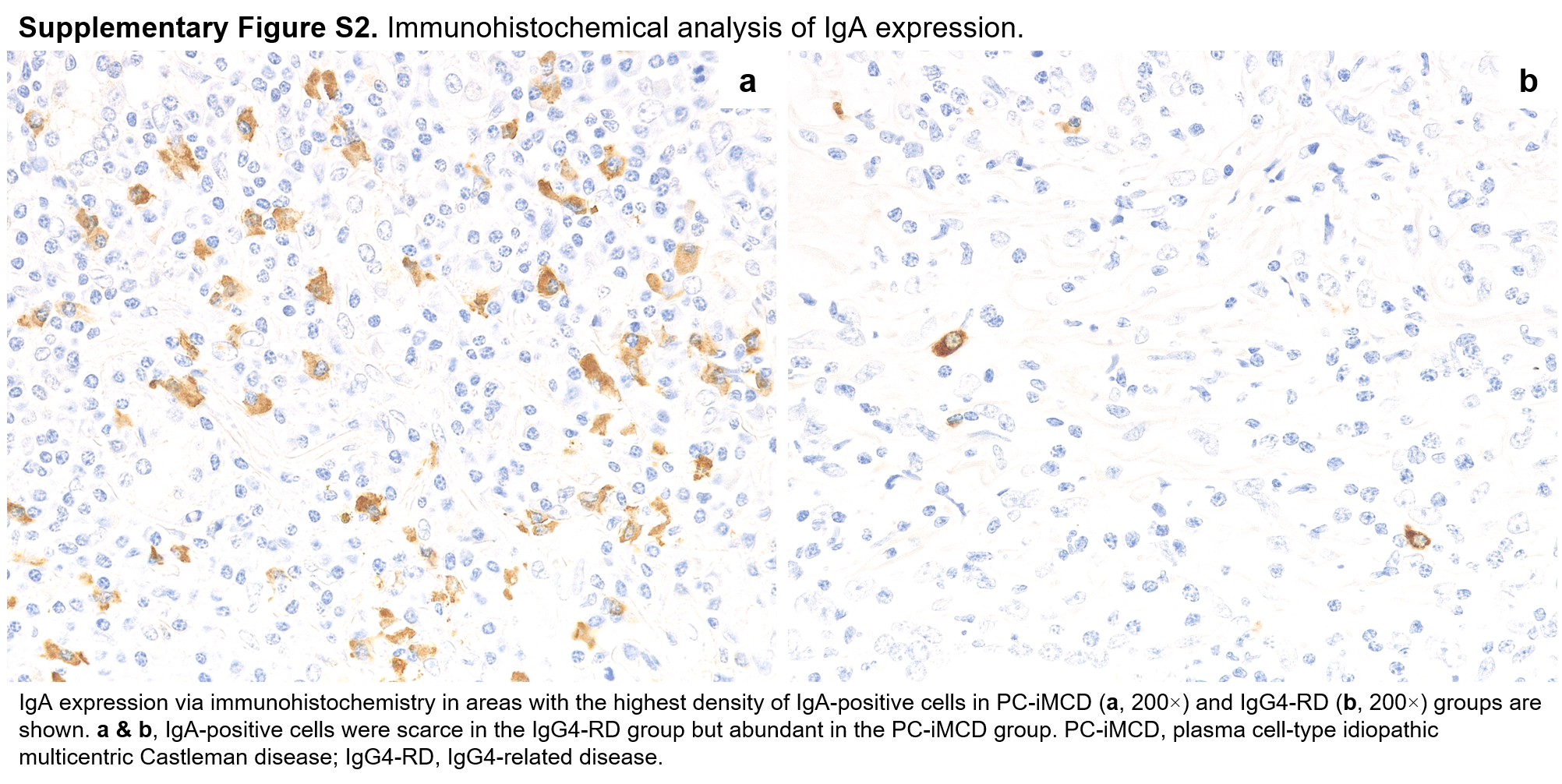

Supplement: Supplementary file 1 [file jpm-10-00269-s001.zip › FigureS2.tif]

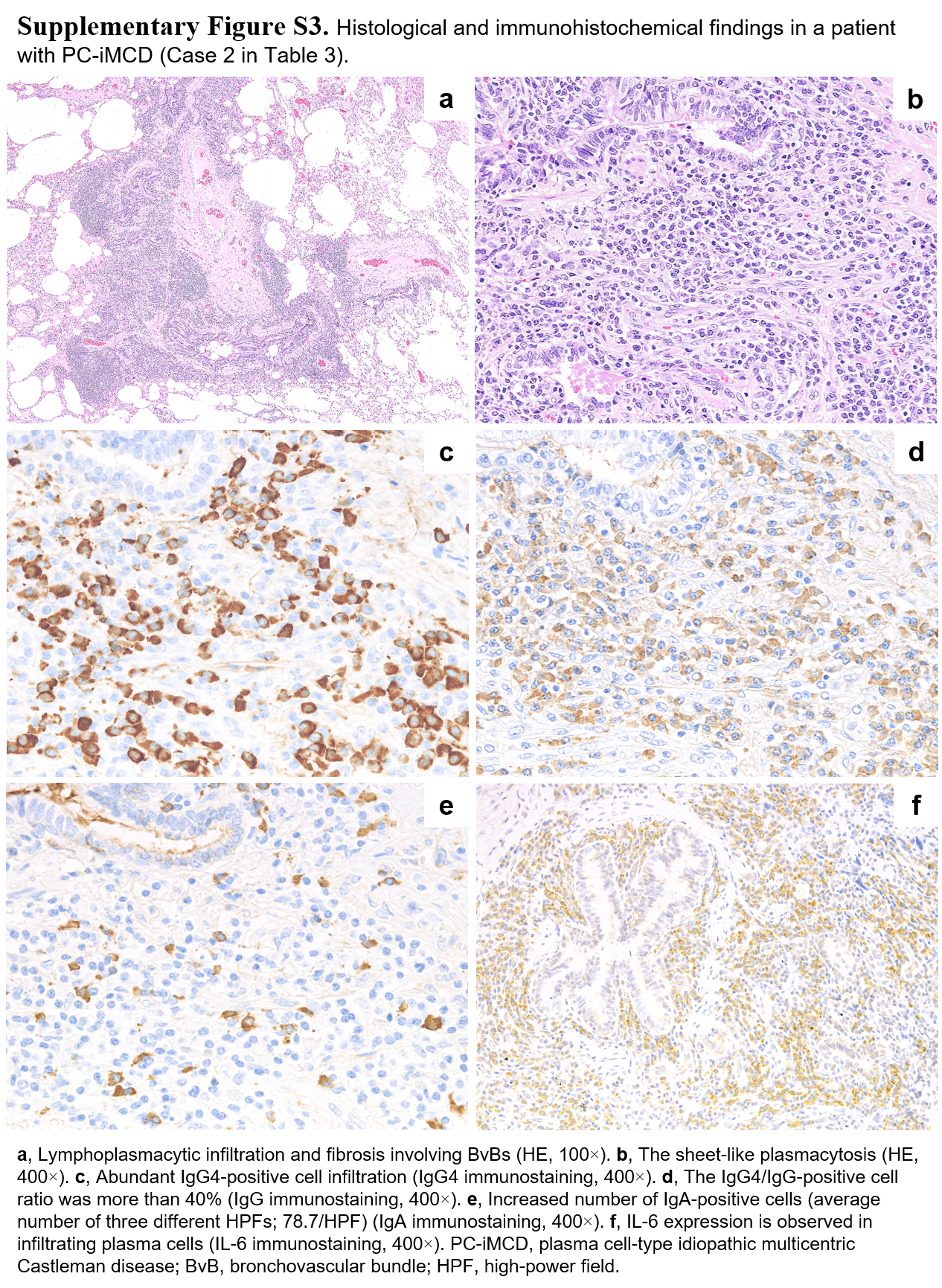

Supplement: Supplementary file 1 [file jpm-10-00269-s001.zip › FigureS3.tif]
